# Evolutionary and Mobile Genetic Element Analysis of a Multidrug-Resistant ST398-MRSA-Vc Isolate from Ready-to-Eat Pork Products

**DOI:** 10.3390/antibiotics15030314

**Published:** 2026-03-19

**Authors:** Jinqi Wan, Xiaoru Wang, Kaifen Wang, Qiuyi Feng, Ruihua Yuan, Xiaojing Qi, Yidong Lai, He Yan

**Affiliations:** 1 College of Food Science and Engineering, South China University of Technology, Guangzhou 510640, China; wchinchi87@163.com; 2 Dongguan Quality Supervision and Testing Center, Dongguan 523808, China; wxru@gddqt.com (X.W.); wkf@gddqt.com (K.W.); fqy@gddqt.com (Q.F.); yrh@gddqt.com (R.Y.); qxj@gddqt.com (X.Q.); 3 Guangdong Province Key Laboratory for Green Processing of Natural Products and Product Safety, Guangzhou 510640, China

**Keywords:** methicillin-resistant *Staphylococcus aureus*, sequence type 398, ready-to-eat food, mobile genetic elements, comparative genomics, One Health

## Abstract

**Background:** Livestock-associated methicillin-resistant *Staphylococcus aureus* (LA-MRSA) ST398 poses a significant zoonotic threat, largely due to its capacity to acquire and disseminate antimicrobial resistance through mobile genetic elements (MGEs). Ready-to-eat (RTE) foods may serve as critical interfaces for zoonotic spillover. However, genomic data on ST398-MRSA-Vc isolates from RTE foods remain scarce, leaving the characteristics of their MGEs largely unresolved. **Methods:** This study performed whole-genome sequencing and comparative genomic analysis of an ST398-MRSA-Vc isolate (NPREF115) from an RTE pork product in China. Using NPREF115 and 134 publicly available *S. aureus* genomes from diverse sources, we constructed a core genome phylogeny and conducted SNP and pangenome analyses, with a focus on MGEs. **Results:** Phylogenetic analysis revealed that our foodborne ST398-MRSA-Vc isolate clustered with human, Capra pyrenaica, bovine, and swine-derived ST398-MRSA-Vc isolates. SNP analysis indicated NPREF115 was most closely related to human clinical isolates (132 and 140 SNPs, respectively), consistent with shared ancestry rather than recent cross-host transmission. Genomic divergence was largely confined to MGEs, including SCC*mec*, prophages, genomic islands, and a chromosomally integrated Tn*560* carrying the *ant*(9)-*Ia*-*lsa*(E)-*lnu*(B) multidrug resistance cluster. Notably, NPREF115 harbored a unique metabolic gene that may facilitate persistence in high-osmolarity food environments. **Conclusions:** The successful colonization of food by the ST398-MRSA-Vc isolate is likely associated with the acquisition of multiple MGEs harboring antimicrobial resistance genes. Transmission of ST398-MRSA-Vc between food, human, and livestock hosts was accompanied by changes in genes involved in metabolism. These findings underscore the importance of monitoring MGEs in genomic surveillance of foodborne MRSA.

## 1. Introduction

Within the One Health framework, food products are increasingly recognized as a potential interface for the transmission of foodborne pathogens between animals and humans. *Staphylococcus aureus*, particularly methicillin-resistant *S. aureus* (MRSA), frequently contaminates meat and other foods during slaughter or processing [1,2,3]. MRSA remains a formidable zoonotic “superbug” and a “high-priority” antimicrobial-resistant (AMR) pathogen responsible for substantial global morbidity and mortality [4,5,6,7]. From a food safety perspective, non-prepackaged ready-to-eat (RTE) foods are of particular public health concern. Sold unpackaged and consumed without further thermal treatment, RTE foods provide favorable conditions for microbial persistence [8]. In China, a 2020 nationwide surveillance by the National Center for Food Safety Risk Assessment (CFSA) detected MRSA in 11.5% of *S. aureus* isolates from RTE foods and beverages [9], underscoring the underappreciated role of the RTE food chain as a silent reservoir for MRSA dissemination.

Among the diverse MRSA lineages, the livestock-associated sequence type 398 (ST398) has emerged as a paradigm of zoonotic transmission [10,11]. While traditionally recognized as an occupational hazard to farmers and veterinarians in Europe and North America [11], ST398 is increasingly detected in retail foods [8], raising concerns about its potential role in foodborne transmission and disease. The capacity of ST398 to persist in diverse environments and cross species boundaries is largely driven by its highly dynamic accessory genome, with mobile genetic elements (MGEs) playing a key role in shaping its phenotypic plasticity, antimicrobial resistance, and host adaptation [12]. Notably, certain ST398 sub-lineages have reacquired human immune evasion clusters (IECs) via prophages or *Staphylococcal* pathogenicity islands (SaPIs), restoring their capacity for sustained human-to-human transmission [13]. Therefore, comprehensive characterization of the MGE repertoire is essential for understanding the evolutionary mechanisms underlying environmental persistence and cross-host adaptation of foodborne ST398.

MRSA has been reported in a wide range of retail foods globally, including RTE products [8,14,15,16,17]. However, genomic characterization of foodborne isolates is still markedly underrepresented compared with isolates from clinical and livestock sources. This knowledge gap is particularly evident for ST398-MRSA from RTE foods, for which systematic whole-genome sequencing data remain limited. Moreover, available genomic investigations have rarely focused on ST398-MRSA-Vc isolates from RTE food matrices, leaving the composition of their MGEs and their roles in food chain transmission largely unresolved. Existing studies on foodborne *S. aureus* in China have largely emphasized epidemiological surveillance. For example, Liu et al. (2023) conducted a genomic epidemiological analysis of *S. aureus* from raw milk in Jiangsu Province and identified ST398 lineages with broader host tropism, highlighting their potential zoonotic transmission risk and public health relevance [18]. Another nationwide investigation of MRSA from RTE foods in China generated valuable data on prevalence, AMR profiles, and virulence gene distribution among foodborne isolates [8]. These findings collectively suggest that the potential public health risks associated with *S. aureus* in foods should not be overlooked. Therefore, genome-resolved investigations are needed to better understand the genetic features, evolutionary relationships, and MGEs of foodborne isolates. To our knowledge, genome-resolved characterization of ST398-MRSA-Vc from RTE products in China has not been systematically reported.

To address this gap and differentiate from broader surveillance studies, we utilized a highly representative ST398-MRSA-Vc isolate (NPREF115) recovered from RTE pork products in China as a high-resolution genomic case study. By performing deep comparative phylogenomics against 134 publicly available *S. aureus* genomes from human-associated, environmental, food, and animal sources, we aimed to (i) characterize the genetic determinants of AMR and virulence, with emphasis on MGEs; (ii) identify the phylogenetic relationships of ST398 across sources and geographic regions; and (iii) explicitly link specific MGEs to environmental persistence in food matrices and evolutionary risks for zoonotic spillover within a One Health context.

## 2. Results and Discussion

### 2.1. Phylogenetic Analysis of ST398-MRSA-Vc Isolate

**Figure 1:**
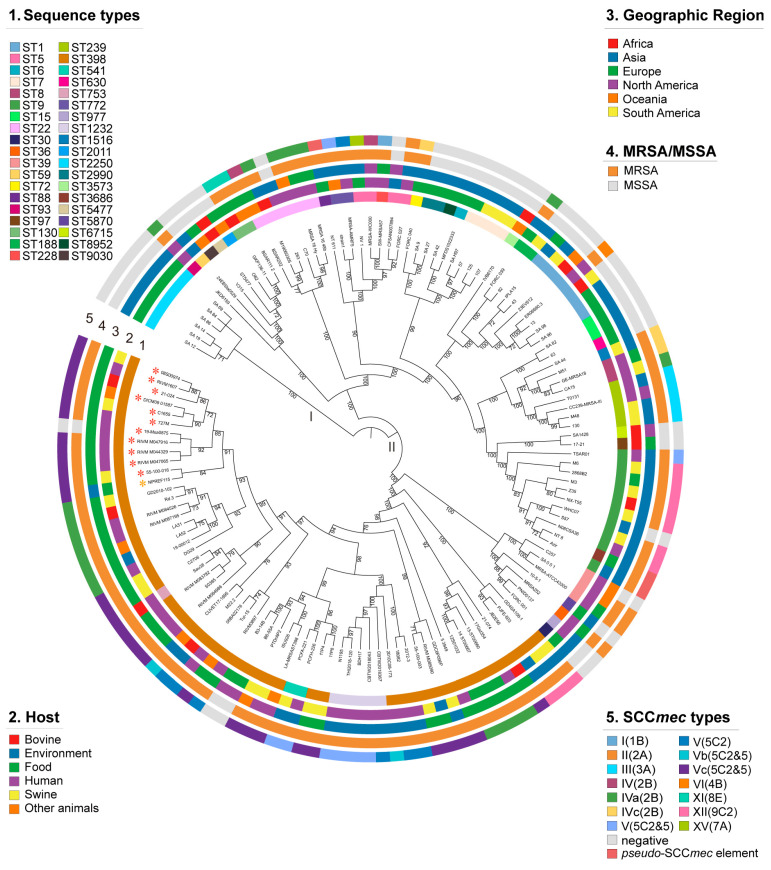
Maximum-likelihood phylogeny of 135 *S. aureus* isolates based on 679 single-copy orthologs. The annotation rings surrounding the tree, from inside to outside, depict (1) sequence types, (2) host, (3) geographic region, (4) MRSA/MSSA, and (5) SCC*mec* type. The strains marked with * belong to Group A. Red asterisks indicate strains retrieved from NCBI, whereas the yellow asterisk indicates the strain sequenced in this study (NPREF115).

We determined a core gene sequence-based phylogeny for our novel isolate and reference isolates, based on 679 single-copy orthologs present in the 135 genome sequences (Figure 1). This tree resolved two lineages (lineages I–II). Phylogenetic lineages exhibited a strong association with sequence types (Figure 1). Most strains of the same STs formed single clades. Lineage I contained one clade predominantly of ST2250. Lineage II encompassed two major clades. One was predominantly composed of ST9, ST1, ST22, ST239, and ST7, and the other was dominated by ST398. The ST398 isolates clustered tightly into a single monophyletic branch representing a typical LA-MRSA lineage, with additional strains assigned to ST1232, ST39, ST541, and other sequence types. All of the 28 ST398-MRSA-Vc isolates appear within a single clade of lineage II. As observed in Figure 1, certain branches exhibited host-specific clustering: the ST398 branch predominantly comprised strains of human and swine origin, consistent with the recognized zoonotic nature of this lineage [4]; the ST22, ST39, and ST1232 branches were mainly composed of human-derived strains. Additionally, a small number of branches showed admixture of environment-derived strains with animal- and human-derived strains, suggesting close genetic relatedness among isolates from different sources.

In the phylogenetic tree (Figure 1), a relatively weak association was observed between phylogeny and geographic origin. Nevertheless, *S. aureus* isolates from distinct geographic regions clustered within the same clade of the whole-genome phylogenetic tree, suggesting frequent interregional migration of isolates between geographic regions, similar to a previous study [12]. Notably, certain subtypes exhibited region-specific clustering, with ST2250 serving as a representative example exclusively found in Asia. This observation suggests that ST2250 may represent a regionally restricted or endemic sequence type. We identified a distinct cluster (group A), which comprised one methicillin-susceptible *S. aureus* (MSSA) strain (19-Msa0875) and 11 ST398-MRSA-Vc strains derived from human, food, Capra pyrenaica, bovine, and swine sources. Within group A, the swine-derived strain 55-100-016 and the food-derived strain NPREF115 were located at the base of the clade. Notably, all remaining isolates in group A, except NPREF115, originated in Europe. This is consistent with recent findings by Cui et al. (2022), who reported that pig-associated MRSA ST398 in China (Qinghai) established a close evolutionary relationship with European and Australian MRSA ST398 [19].

### 2.2. Biological Features of S. aureus Isolates

**Figure 2:**
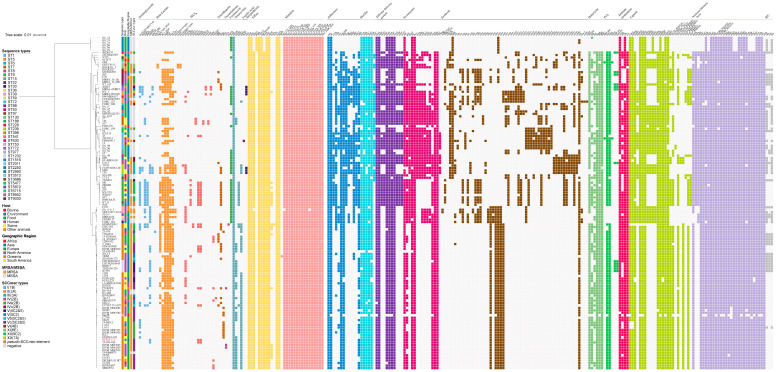
Virulence and antibiotic resistance profiles across a phylogeny of 135 *S. aureus* isolates based on single-copy core orthologs. Single-copy orthologous genes were found using OrthoFinder version 2.4.0. The pattern of gene presence (colored blocks) or absence (white) is shown. The presence/absence gene matrix represents, from left to right, antibiotic resistance genes involved in aminoglycoside, beta-lactam, MLS_B_, trimethoprim, oxazolidinone, fosfomycin, tetracycline, fusidic acid, streptothricin, efflux, mutation, virulence genes involved in adherence, biofilm, effector delivery system, exoenzyme, exotoxin, hemolysin, PVL, immune modulation, capsule, nutritional/metabolic factors, and IECs. The strain highlighted in red was sequenced in this study (NPREF115).

Variations in virulence and AMR gene profiles were observed among *S. aureus* lineages and subgroups (Figure 2). With respect to the association between STs and AMR genes, ST9 strains consistently carried multiple aminoglycoside resistance genes (*aac*(6′)-*Ie/aph*(2″)-*Ia*, *aadD1*, *ant*(6)-*Ia*) and MLS_B_ resistance genes *(lnu*(B), *lsa*(E)), as well as the trimethoprim resistance gene *dfrG* and tetracycline resistance gene *tet*(L). This concentrated co-occurrence pattern indicates that ST9 strains commonly harbor a high burden of AMR determinants. The classical co-carriage of *ant*(9)-*Ia* and *erm*(A), typically associated with transposon Tn*554*, was retained in isolates belonging to ST1232, ST239, ST5, and ST39, as well as in a subset of the ST398 lineage. Notably, within the ST398 lineage, a distinct subpopulation harbored a specific resistance profile consisting of *ant*(9)-*Ia*, *lsa*(E), and *lnu*(B). The linkage between *lsa*(E) and *lnu*(B) has been well documented [20,21,22], as these genes are frequently co-localized within multidrug resistance gene clusters. Mechanistically, *lsa*(E) encodes an ATP-binding cassette F (ABC-F) protein conferring cross-resistance to pleuromutilins, lincosamides, and streptogramins [23], whereas *lnu*(B) encodes a lincosamide nucleotidyltransferase. Macrolides and lincosamides are key antibiotics for the treatment of common infections in livestock [24], and spectinomycin is usually used in combination with lincomycin as a fixed therapy. The coexistence of their corresponding resistance genes highlights a specific evolutionary adaptation of this ST398 subgroup under veterinary antimicrobial selection pressure, which is consistent with the findings of Jiang et al. (2024) [25].

In our study, all ST398-MRSA-Vc isolates harbored 4–8 classes of AMR genes, which collectively mediate the characteristic resistance spectrum of this clone. NPREF115 carries the *mecA* (and its associated regulatory genes), together with additional AMR determinants including *blaZ*, *ant*(9)-*Ia*, *lsa*(E), *lnu*(B), *tet*(K), and *tet*(M). Consistent with this genetic profile, the isolate exhibited phenotypic resistance to oxacillin/cefoxitin, tetracycline, and clindamycin. However, phenotypic streptomycin susceptibility in our isolate was inconsistent, given the presence of *ant*(9)-*Ia*, which was consistent with another study [26]. Analysis of virulence genes revealed that most non-ST398 strains harbored a greater number of virulence genes than ST398 strains, particularly genes associated with exotoxins. However, the exotoxin genes *set1* and *set3*–*set6* were enriched in ST398 strains. Notably, within the ST398 lineage, only three strains (2010C08-173, 18082, and 17Gst354) carried an immune evasion cluster (IEC, containing *chp*, *sak*, and *scn*) and concurrently lacked the *tet*(M) gene, a genetic profile characteristic of the human-adapted CC398 clade [27].

### 2.3. Phylogenetic Analysis Based on Core Genome SNPs

**Figure 3:**
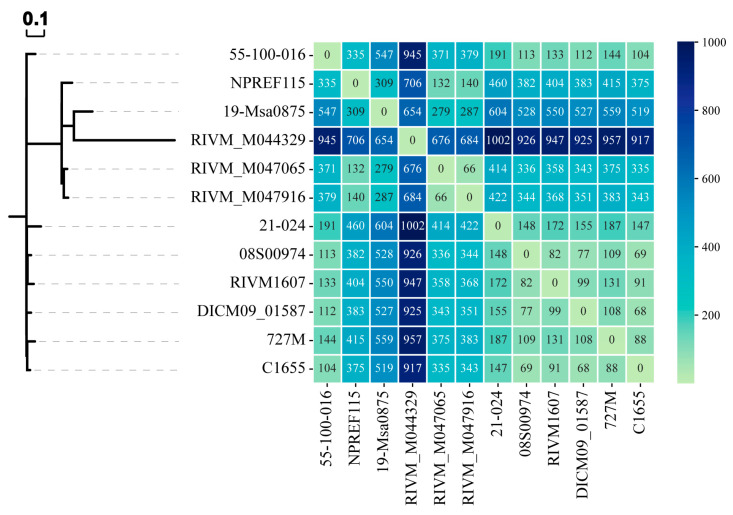
Core genome SNPs phylogenetic analysis of NPREF115 and its relative strains. The phylogenetic tree was constructed based on core-genome SNPs, with the NPREF115 genome as reference. The values in the matrix denote the pairwise SNP differences between strains, reflecting the number of single-nucleotide substitutions across their core genomes.

SNP analysis offers high accuracy and resolution for distinguishing strains within the same evolutionary lineage [28]. Using strain NPREF115 as the reference, a pairwise SNP distance matrix derived from the core genome alignment was generated for 6 human-derived, 3 swine-derived, 1 bovine-derived, and 1 Capra pyrenaica-derived *S. aureus* strains within the same subclade, and a phylogenetic tree was constructed based on this matrix (Figure 3). The results showed that strain NPREF115 clustered with four human-derived strains, including one MSSA strain (19-Msa0875), indicating the presence of both methicillin-resistant and -susceptible strains within the same ST398 clade. The intercalation of MSSA within an MRSA-dominant clade highlights the instability of the SCC*mec* element in this lineage.

From a fitness trade-off perspective, while SCC*mec* confers a critical survival advantage under antibiotic pressure, maintaining this large genomic island imposes a significant metabolic burden. As observed by Huber et al. (2022) in the ST398 lineage, in environments where β-lactam selective pressure is relaxed, bacteria may dismiss the fully replicative SCC*mec* element through homologous recombination to save energy [29]. This reduction in metabolic burden allows the pathogen to better adapt to livestock-associated environmental stressors, including high ammonia concentrations and contact with host-specific proteins. Such an evolutionary strategy of spontaneous SCC*mec* loss enables the pathogen to rapidly mitigate fitness costs when antibiotic selection is absent, thereby maintaining population competitiveness in complex environments. These findings are consistent with recent research by Scharn et al. (2022), which demonstrated that SCC*mec* elements can undergo spontaneous excision when antibiotic selective pressure is removed [30].

In the phylogenetic tree (Figure 3), the swine-derived strain 55-100-016 formed a distinct branch, suggesting possible host-specific adaptation or an independent evolutionary trajectory. Additionally, two other clades, each comprising one human-derived and two livestock-associated isolates, demonstrated high genomic similarity across host origins. Furthermore, NPREF115 showed the fewest SNP differences between two SCC*mec* Vc-type isolates (RIVM_M047065 and RIVM_M047916) recovered from human nasal samples in the Netherlands, with 132 and 140 SNPs, respectively. The next closest isolates were the MSSA strain 19-Msa0875 from a human nasal sample in Switzerland and the SCC*mec* Vc-type strain 55-100-016 from a swine sample in Denmark, differing by 309 and 335 SNPs, respectively. All 12 isolates analyzed in this study belonged to sequence type 398. Genomic characterization further revealed a consistent profile across these strains: the absence of the IEC marker (*scn*-negative) and the presence of the tetracycline resistance gene (*tet*(M)-positive). The IEC (*chp*, *sak*, and *scn*) genes are thought to be a marker of human adaptation and are rarely present in LA-MRSA [31,32]. The combination of this specific genotype within the ST398 clonal background definitively classifies these strains as the classic livestock-associated lineage, distinct from the human-adapted clade [27]. This genetic background in clinical strains argues against long-term human adaptation, consistent with the definition of spillover transmission, where the pathogen enters a novel host without acquiring the necessary adaptive traits for sustained transmission [33]. Although NPREF115 clustered phylogenetically with several human-derived isolates, the uniform retention of the canonical livestock-associated genotype (*scn*-negative, *tet*(M)-positive) across this sub-lineage does not support stable human adaptation. Overall, the available evidence is more consistent with a livestock-associated origin for NPREF115 and related clinical isolates.

High-resolution SNP analysis further showed that NPREF115 differed by 132 and 140 core genome SNPs from two SCC*mec* Vc-type clinical isolates from the Netherlands. Using the conservative cutoffs of 25 wgSNPs proposed by Coll et al. (2020), above which transmission of MRSA within the previous 6 months can be ruled out [34], we can exclude possible transmission events between the closest strains (NPREF115, RIVM_M047065, and RIVM_M047916) within 6 months. This strain pair does not comply with the criteria established for the occurrence of direct transmission (<25 SNPs) because the number of SNP differences greatly exceeds thresholds. Instead, these values are consistent with shared ancestry within a circulating European ST398 sub-lineage rather than short-term epidemiological linkage.

### 2.4. Comparative Whole-Genome Analysis of NPREF115 and Closely Related ST398 Isolates

**Figure 4:**
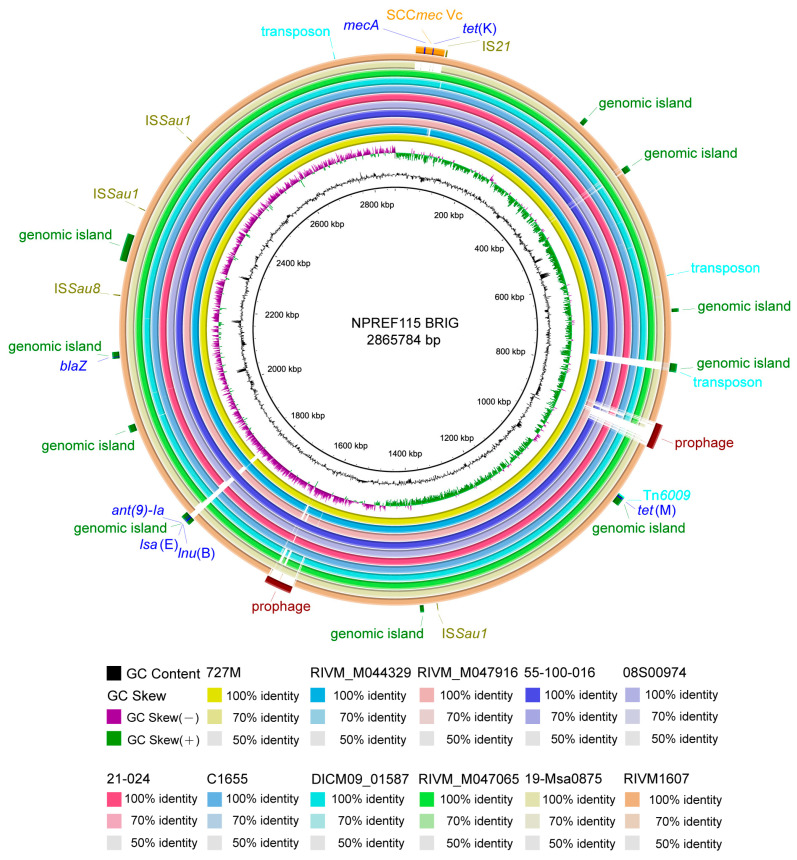
Ring comparison of NPREF115 using BRIG. Orange represents the SCC*mec* element, green indicates genomic islands, brown denotes prophages, olive corresponds to insertion sequences, and sky blue marks transposons. Resistance genes are depicted in blue.

To investigate genomic variation, a whole-genome comparison was performed using BRIG, with NPREF115 as the reference (Figure 4). Pairwise ANI analysis showed that NPREF115 shared 99.87–99.93% nucleotide identity with the 11 related ST398 isolates (Supplementary Table S3), confirming the high genomic conservation within this sub-lineage. The regions of divergence were primarily restricted to MGEs, including the SCC*mec* element (33,751–80,304 bp), two prophage regions (872,123–917,560 bp and 1,606,024–1,650,802 bp), and three genomic islands (435,446–445,302 bp, 771,839–785,833 bp, and 1,803,238–1,821,212 bp). A detailed summary of the identified MGEs in NPREF115, including their genomic coordinates, sizes, and associated resistance or virulence genes, is provided in Supplementary Table S4. The results indicate that almost all ARGs reside on MGEs, such as *mecA*, *tet*(K), *tet*(M), *ant*(9)-*Ia*, *lsa*(E), *lnu*(B), and *blaZ*.

Pan-genome analysis of the 12 ST398 isolates identified 3105 gene families, of which 2382 (76.7%) were classified as core genes present in all genomes, while 723 (23.3%) constituted the accessory genome. The accessory genome was enriched in MGEs and accounted for most inter-strain variability, consistent with the localized divergence observed in the BRIG comparison. Despite diverse host origins and geographic backgrounds, the ST398 isolates therefore maintained a highly conserved core genomic scaffold, suggesting limited chromosomal diversification within this sub-lineage. These findings collectively indicate that HGT primarily shapes the accessory genome, while the core genome remains relatively stable across different host and source backgrounds, consistent with the observations reported by Jamrozy et al. (2017) [35].

Compared with the remaining 11 ST398 isolates, three genes were unique to NPREF115, encoding a putative lipid kinase, a hypothetical protein, and the 5-oxoprolinase subunit A. Functional annotation indicated that these genes are associated with metabolic processes. Among these, the presence of a gene encoding 5-oxoprolinase is of particular interest. 5-oxoprolinase catalyzes the conversion of 5-oxoproline to glutamate, a metabolite involved in cellular osmotic regulation [36]. Although no functional assays were performed in this study, the acquisition of this gene may confer potential metabolic adaptability under hyperosmotic conditions characteristic of processed meat products. Future studies will focus on the experimental validation of this potential role. Taken together, the identification of strain-specific metabolic genes in NPREF115 raises the possibility that these features may facilitate persistence in anthropogenic food-processing environments.

### 2.5. The Genetic Context of Prophage NPREF115-1

Genome analysis of the food-derived strain NPREF115 revealed a complete prophage with canonical integration and structural gene modules. The prophage region was 45,438 bp in length, with a G + C content of 35.73%. A total of 223 open reading frames (ORFs) were predicted within this region. No antibiotic resistance genes or additional virulence determinants were detected within the prophage region (Figure S3). This finding aligns perfectly with recent genomic epidemiological observations; while prophages are primary vehicles for virulence genes in IEC-positive *S. aureus* lineages, prophages in IEC-negative ST398 isolates typically lack these virulence determinants [18]. Furthermore, similar to previous reports [37], this prophage does not appear to serve as a reservoir for antibiotic resistance genes.

**Figure 5:**
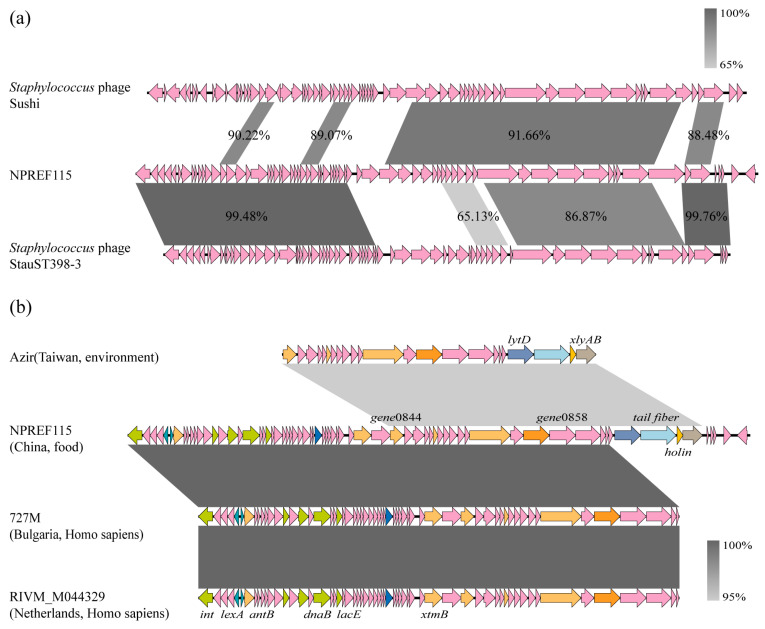
(**a**) Comparative genomic alignment of the prophage region in strain NPREF115 with *Staphylococcus* phages Sushi and StauST398-3. (**b**) Comparative alignment of the prophage identified in strain NPREF115 with homologous genomic regions retrieved from NCBI reference genomes. Arrows represent predicted ORFs, with arrow direction indicating transcriptional orientation. Colors are used for visual distinction and do not necessarily indicate functional categories unless specified.

Despite lacking typical accessory genes, comparative structural analysis revealed a strikingly chimeric architecture shaped by extensive recombination (Figure 5a). The prophage displays a modular organization: the lysogeny/integration module and holin/lysis segment share >99.4% nucleotide identity with *Staphylococcus* phage StauST398-3, whereas the central structural module (encoding capsid and tail components) shows 91.7% nucleotide identity to *Staphylococcus* phage Sushi. Similar to the novel SCC*mec* variant recently identified in LA-MRSA isolates from pig farms in South Korea, which was formed by recombination of distinct functional modules derived from SCC*mec* type V and type XII [38], the prophage characterized in this study also exhibited a mosaic genomic structure. Its lysogenic/integration module and structural module were derived from different phage lineages, respectively. This observation is consistent with previous reports that the mobilome of *S. aureus* exhibits a high degree of modularity and genetic plasticity [39].

Given the mosaic architecture of the prophage identified in NPREF115, we further investigated whether its constituent genomic modules exhibit broader evolutionary dispersion. Comparative searches against NCBI genomes revealed that environmental and human-associated isolates from geographically disparate regions, including East Asia and multiple European countries, carry chromosomal segments showing high nucleotide identity (95–100%) to individual NPREF115 modules (Figure 5b). Notably, phage structural analysis identified a highly conserved genomic region shared with the environmental strain *S. aureus* Azir (gene0844–gene0866). According to PHASTER annotation, this 23-gene cluster constitutes a complete phage structural module encoding head morphogenesis proteins (gene0844–0849), tail components (gene0850–0857), and a lysis cassette (gene0865, holin). This region also contains several host-interaction determinants. KEGG annotation identified gene0858 as a trimeric autotransporter adhesin (TAA), a protein family known to mediate bacterial adhesion to host tissues and abiotic surfaces. In addition, gene0864 was annotated as a tail fiber protein.

The detection of homologous prophage remnants in the phylogenetically distant strain Azir (ST9, environmental origin, Taiwan) provides a broader evolutionary context. ST9 is the main livestock-associated lineage in China, distinct from the LA-MRSA-ST398 lineage dominant in Europe and North America [40]. The detection of homologous prophage remnants in the ST9 strain Azir suggests that related phage modules are distributed across distinct clonal complexes. The cross-lineage exchange of these critical surface-binding determinants between the locally dominant ST9 and the introduced ST398 suggests potential HGT between distinct clonal lineages, enabling disparate clones to acquire convergent colonization capabilities within the same ecological reservoir. This observation is strongly supported by recent findings in Korean pig farms, where the emergence of novel SCC*mec* variants and the ongoing clonal expansion of CC398 were linked to the intricate infrastructure of breeding networks [38]. Such networks likely serve as environmental conduits that facilitate the dissemination and recombination of MGEs, reinforcing the role of livestock production chains as dynamic arenas for the genetic diversification of LA-MRSA.

Current evidence indicates that phage-mediated host lysis is primarily driven by the holin–endolysin system [41]. Holins are pore-forming proteins that permeabilize the bacterial cytoplasmic membrane and thereby precisely control the timing of lysis. Endolysins are enzymatic proteins responsible for degrading the bacterial cell wall (peptidoglycan). Together, these proteins lyse the host bacteria from the inside, complete the lytic cycle, and promote the release of progeny phages. A detailed comparison of the terminal region of the prophage revealed a key difference between NPREF115 and the clinical strains (RIVM_M044329/727M). While NPREF115 retains the downstream region, including the tail fiber and holin genes (gene0864–gene0865), the clinical strains exhibit a truncation after gene0861. The absence of the holin-containing region in clinical strains may reduce the potential for lytic induction, although experimental validation would be required to confirm functional consequences. In contrast, the intact module in NPREF115 indicates preservation of structural and lysis-associated genes. Whether this difference has functional consequences remains to be determined.

The specific loss of the lytic module (holin/tail fiber) in clinical strains is consistent with the evolutionary theory of prophage domestication. According to the framework proposed by Bobay et al. (2014), intact prophages may pose a fitness cost to bacterial hosts upon induction of the lytic cycle [42]. The selective deletion of lysis-associated genes observed in the clinical strains could be consistent with progressive prophage degradation. In contrast, NPREF115 retains a structurally complete prophage region. However, without temporal or experimental evidence, it is not possible to determine whether this represents a recent acquisition event or differential retention under distinct environmental conditions. These observations are based on comparative genomic analyses and warrant further validation through temporal and functional investigations.

### 2.6. Identification and Characterization of a Putative Cryptic ICE Encoding a Functional T4SS

Functional annotation revealed that this genomic island represents a putative cryptic ICE that utilizes a Type IV Secretion System (T4SS) for horizontal transfer. Structural analysis identified a complete conjugation module comprising a NicK-family relaxase (transfer initiation) and a VirB4-like ATPase (assembly energization), together with an FtsK translocase and a TcpD/E membrane scaffold complex. Notably, the element encodes a CwlO-like peptidoglycan hydrolase, likely facilitating the penetration of the thick Gram-positive cell wall during conjugation. The island is flanked downstream by △IS*Sau5* (IS*30* family transposase) remnants that mark the recombination boundary formed upon integration into the host chromosome.

**Figure 6:**
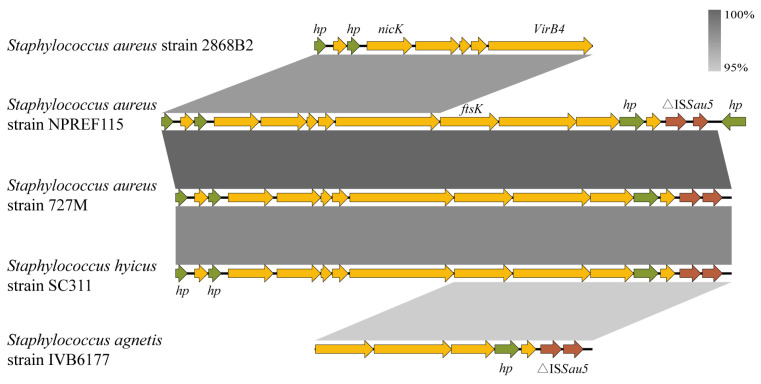
Schematic presentation of the genetic context of the genomic island in the chromosomal DNAs of the isolate NPREF115 compared with the corresponding regions of other strains in the NCBI database. ORFs are shown as arrows, indicating the transcription direction, and the colors of the arrows represent different fragments. Brown represents the insertion sequence. Olive color represents the hypothetical protein. Proteins with other functions are shown in yellow. The △ symbol represents the truncated genes.

Comparative genomic analysis demonstrated that this ICE is embedded within a conserved syntenic module broadly distributed across diverse *Staphylococcus* species (Figure 6). Although NPREF115 was isolated from an RTE pork product, its genomic island displayed nearly complete nucleotide identity with that of a Bulgarian clinical isolate (strain 727M; 100%), with both belonging to the ST398-SCC*mec* Vc lineage. Substantial conservation was also observed with MRSA strain 2868B2 (ST9-SCC*mec* XII) recovered from frozen dumplings sourced from Hangzhou (96.99% identity over 47.6% coverage), indicating retention of the genomic architecture across divergent *S. aureus* clonal backgrounds. Strikingly, the same syntenic structure extended beyond *S. aureus*: the swine-associated strain *Staphylococcus hyicus* SC311 maintained high nucleotide identity (98.07%), and a more diverged but clearly homologous region remained detectable in *Staphylococcus agnetis* IVB6177 isolated from camel nasal swabs (93.49%). Collectively, these data suggest that this ICE is widely distributed across distinct *staphylococcal* lineages and host species.

The genomic island identified in this study represents a “cryptic” ICE: although it has a fully functional junction mechanism (relaxase, T4SS, and LysM/CwlO modules), it lacks classical virulence or antibiotic resistance cargo genes. The existence of energetically costly components such as the VirB4 ATPase and FtsK translocase suggests that maintaining this element imposes a significant metabolic burden [43,44], implying that its persistence necessarily confers a selective advantage, although the nature of this advantage remains unclear. We propose that this element functions as a “genetic chassis” or carrier. The presence of flanking IS*30*-family elements (△IS*Sau5*) indicates that this region acts as a recombination hotspot. Since the IS*30* family plays a pivotal role in the insertion of various MGEs [45,46], the genomic islands in NPREF115 and 727M may serve as a preferred locus for the recruitment of additional genetic cargo. Although there is no cargo gene at present, this element retains the capacity to acquire resistance determinants (e.g., *tet*(M) or *cfr*) from its genomic environment, thereby acquiring the potential to serve as a vector for multidrug resistance dissemination.

The identification of this element in *S. aureus*, *S. hyicus*, and *S. agnetis* underscores its ability to cross species barriers. This broad host range is likely facilitated by the encoded CwlO peptidoglycan hydrolase identified in our structural analysis. CwlO is a D, L-endopeptidase that is widely present in Gram-positive bacteria. It primarily functions by cleaving the peptide cross-links within the peptidoglycan to remodel the bacterial cell wall [47]. As the cell wall composition varies slightly among *Staphylococcal* species, the carriage of a dedicated, intrinsic lysis module allows the ICE to breach the peptidoglycan layer of diverse recipients, independent of host-specific autolysins.

The almost identical ICE in the Chinese foodborne isolate NPREF115 and a Bulgarian clinical ST398 strain provides evidence for the intercontinental dissemination of LA-MRSA ST398 via interconnected livestock, food, and human pathways. Despite the lack of resistance genes, the conservation across animal, food, and human staphylococci highlights the importance of cryptic MGEs as silent yet potentially high-risk vectors for the future dissemination of AMR within the One Health framework.

### 2.7. Genetic Environment of the Multidrug Resistance Transposon Tn560 in Chromosomal DNA

Bioinformatic analysis revealed that strain NPREF115 harbors the transposon Tn*560*, a recently described derivative of the Tn*554* family [48]. This element is site-specifically integrated at the 3′ end of the chromosomal DNA repair gene *radC*, a known integration hotspot within the CC398 lineage. The *radC* locus has previously been shown to accommodate diverse transposable elements in LA-MRSA isolates [49,50]. Structurally, the identified element displays the characteristic organization of the Tn*554* family, retaining the core transposase module (*tnpA*, *tnpB*, and *tnpC*). However, it differs markedly from classical Tn*554*, which typically harbors the MLS_B_ resistance gene *erm*(A) and is predominantly associated with European phylogroups [49]. In contrast, Tn*560* in NPREF115 has undergone cargo substitution, lacking *erm*(A) and instead harboring the *lsa*(E)-*lnu*(B) resistance cluster. The nucleotide sequence of this element shares 99.95% identity with the prototype Tn*560* reported in the porcine MRSA strain GDC6P096P (GenBank accession no. MW832219) [48], thereby confirming its identity. Furthermore, this genetic backbone showed 99.99% nucleotide identity with that of the swine-derived *S. aureus* strain 55-100-016.

**Figure 7:**
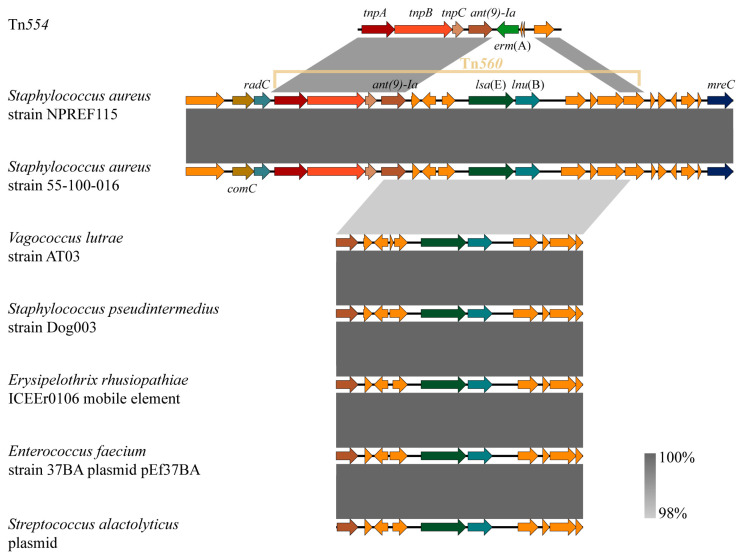
Comparison and homology analysis of the *lsa*(E) gene environment in different bacterial strains. The arrows indicate the directions of gene transcription, and different genes are represented by different colors.Orange arrows indicate hypothetical proteins. Colored arrows represent genes with predicted functions, including antimicrobial resistance genes (labeled), transposition-related genes (e.g., *tnpA*, *tnpB*, *tnpC*), and other annotated functional genes. Areas shaded grey represent regions of >98% nucleotide sequence identity.

Comparative genomic analysis (Figure 7) unveiled a notable evolutionary pattern: while the Tn*554* transposase machinery appears specific to *S. aureus*, the internal resistance module (*ant*(9)-*Ia*-*lsa*(E)-*lnu*(B)) is highly conserved across phylogenetically diverse taxa. Cross-species comparison further showed that the homologous *ant*(9)-*Ia*-*lsa*(E)-*lnu*(B) cluster is present in multiple Gram-positive taxa, including *Staphylococcus pseudintermedius*, *Vagococcus lutrae*, and *Erysipelothrix rhusiopathiae* (embedded within ICE elements), as well as in plasmids of *Enterococcus faecium* and *Streptococcus alactolyticus*, each exhibiting >98% sequence identity. Notably, these non-*S. aureus* species harbor the resistance cluster without the flanking Tn*554* structural framework, supporting a model of horizontal module exchange. Our data strongly support the hypothesis that Tn*560* originated through interspecies recombination between a Tn*554* progenitor and an *enterococcal* plasmid [45]. Specifically, the *ant*(9)-*Ia*-*lsa*(E)-*lnu*(B) module in NPREF115 shows >98% sequence identity to plasmid-borne clusters prevalent in *Enterococcus* species. Previous studies have documented that *lsa*(E) is widespread among animal-associated *Enterococcus* and is typically located on MGEs amenable to plasmid-mediated transfer [21,51]. This striking structural conservation between *enterococcal* plasmids and the *staphylococcal* genomic island suggests that the ancestral Tn*560* likely captured this resistance module from the *enterococcal* gene pool, which represents a primary reservoir of pleuromutilin resistance [52]. Subsequently, integration into the *radC* hotspot via Tn*554*-derived machinery enabled stable chromosomal fixation [49], thereby allowing vertical inheritance of the MDR phenotype even in the absence of direct antimicrobial selection.

The *lsa*(E) gene encodes an Lsa-type ABC-F protein that confers combined resistance to PLSA antimicrobials, which play important roles in the prevention and treatment of bacterial diseases in both humans and animals [23,53]. Although *lsa*(E) has been detected in *S. aureus* from human [54], bovine [55], porcine, and poultry sources [21,56], our study reports its presence in a foodborne isolate, underscoring its entry into the food chain. Surveillance data from China suggest that most *lsa*(E)-positive foodborne *S. aureus* strains are epidemiologically linked to livestock or poultry production systems, with evidence of transmission between animals and food products [57]. In this study, *lsa*(E) and *lnu*(B), which mediate resistance to lincosamides by encoding lincosamide nucleotide transferases [58], co-occurred in the isolate (Figure 7). This observation is consistent with previous reports showing that *lsa*(E) and *lnu*(B) are typically located in close proximity and are often co-inherited and co-transferred [20,21,22]. The *ant*(9)-*Ia* gene that mediates spectinomycin resistance was found upstream of *lsa*(E) in strain NPREF115, forming a tripartite arrangement (*ant*(9)-*Ia*-*lsa*(E)-*lnu*(B)). Critically, chromosomal integration of this MDR cluster within Tn*560* eliminates the instability associated with plasmid-borne resistance, effectively locking the resistance phenotype into the genome. Given that *lsa*(E) often resides within a broader multidrug resistance island that includes multiple ARGs [52,58,59,60], *lsa*(E)-positive isolates may therefore serve as reservoirs for the accumulation and dissemination of additional ARGs. Collectively, these findings highlight a significant food safety and public health concern: the emergence of chromosomally stabilized, multidrug-resistant *S. aureus* clones carrying *lsa*(E) in the food supply. Continuous surveillance of *lsa*(E) and its genetic contexts in foodborne and livestock-associated *S. aureus* is therefore imperative to mitigate the risk of resistance spread across the One Health framework.

Accumulating evidence indicates that swine populations constitute a major reservoir of ST398, with multiple studies from China reporting its widespread occurrence in pig production systems, including farms and slaughterhouses [19,40]. This livestock association provides an ecological foundation for the persistence and dissemination of ST398 within animal production environments. Beyond the animal reservoir, increasing reports have documented the emergence of ST398 in human colonization and infection cases. Notably, surveillance studies from Taiwan revealed that most locally circulating ST398 isolates belonged to human-adapted lineages carrying IEC genes, although sporadic livestock- and pork-associated isolates were also identified [61]. At the food chain level, recent international investigations further support the role of pork production systems in facilitating ST398 dissemination. For example, a slaughterhouse-based study in Portugal demonstrated widespread ST398 contamination across live pigs, processing environments, retail meat products, and workers, with whole-genome SNP analysis supporting multiple cross-transmission events along the production continuum [62]. Similarly, Spanish surveillance of pig-derived food products identified CC398 as the predominant lineage in pork, with most MRSA-CC398 isolates exhibiting tetracycline resistance and lacking IEC genes, a genetic profile consistent with a livestock-associated background [63]. Within China, large-scale investigations of foodborne *S. aureus* associated with food poisoning events between 2011 and 2021 revealed high genetic diversity, with CC398 emerging as a predominant lineage and showing close links to cross-host transmission between livestock and humans [64]. Together, these findings indicate that contamination of food products with ST398 is unlikely to represent isolated events but rather reflects a dynamic livestock–food–human transmission interface.

In this context, the recovery of ST398-MRSA from RTE pork in the present study provides additional genomic evidence supporting a potential transmission continuum linking livestock, food products, and human exposure. Although direct transmission cannot be conclusively inferred from genomic data alone, the high core genome conservation observed among the isolates, together with the enrichment of MGEs in the accessory genome, suggests that HGT may contribute to the adaptive success and ecological fitness of this lineage. Thus, the potential public health concern highlighted here is more closely linked to the mobility of resistance genes carried by MGEs than to confirmed clonal spread in human populations. From a One Health perspective, these findings underscore the importance of integrated surveillance across animal, food, and human sectors. RTE foods, which bypass further cooking prior to consumption, may represent a particularly relevant yet underrecognized vehicle for community exposure to MRSA. Continued genomic surveillance and source-tracking efforts will be essential to clarify transmission pathways and to inform targeted risk mitigation strategies at the human–animal–food interface. However, this study is based on a single newly sequenced foodborne ST398 isolate, and therefore, the prevalence, genetic diversity, and transmission dynamics of this lineage in Chinese RTE products cannot be determined from the present data. While comparative analysis with publicly available genomes provides important evolutionary context, broader and systematic surveillance is required to assess the extent of dissemination and public health significance of this isolate.

## 3. Materials and Methods

### 3.1. Isolates

From June 2021 to September 2025, we collected 1917 samples of non-prepackaged RTE foods and 925 processing environment samples in Dongguan, Guangdong Province, including school-enterprise canteens, restaurants, supermarket RTE food counters, farmers’ markets, and mobile food vendors, covering most of the urban and rural areas of the city. A total of 54 *S. aureus* strains were isolated from these sampling sites, including 50 from food samples and 4 from environmental samples. These foodborne strains were isolated from nine types of food products, including 18 isolates from cooked meat products, 13 isolates from cooked noodle and rice products, 9 isolates from snacks, 3 isolates from fruit and vegetable products, 2 isolates from hot dishes, 2 isolates from cold vegetable dishes in sauce, 1 isolate from salad, 1 isolate from fresh-cut fruit, and 1 isolate from RTE raw seafood. All the isolates were obtained according to GB 4789.10-2016, Food Microbiological Examination of *S. aureus* (National Food Safety Standards of China) [65].

The ST398 MRSA isolate (NPREF115) analyzed in this study was isolated in 2022 from an RTE pork product obtained from a restaurant in Dongguan, Guangdong Province, China. Susceptibility to 19 antimicrobial agents was determined using the disc agar diffusion method and the E-test method. The isolate displayed resistance to multiple antibiotics, including oxacillin, cefoxitin, penicillin, nitrofurantoin, tetracycline, minocycline, and clindamycin (Supplementary Table S1).

### 3.2. Genome Sequencing, Assembly, and Annotation

Genomic DNA was extracted from an overnight culture grown in a brain/heart infusion broth at 37 °C using the Bacterial DNA extraction kit (magnetic beads) (Majorbio, Shanghai, China). Purified genomic DNA was sequenced using the PacBio Sequel IIe (Pacific Biosciences, Menlo Park, CA, USA) and Illumina NovaSeq 6000 (Illumina, San Diego, CA, USA) platforms. Hybrid assembly was performed using Unicycler v0.4.8. The final assembly generated a single circular chromosome of 2,868,776 bp with a GC content of 32.98%. The average sequencing depth was 87.31×, and the PacBio long-read N50 was 9376 bp, supporting high assembly completeness and accuracy (Supplementary Figure S1). The complete genome sequence of the MRSA strain NPREF115 has been deposited in GenBank: chromosome (GenBank ID: CP187946) and plasmid (GenBank ID: CP187947).

Open reading frames (ORFs) were predicted with Glimmer (http://ccb.jhu.edu/software/glimmer/index.shtml, accessed on 15 February 2025), GeneMarkS v4.3, and Prodigal v2.6.3. Functional annotation of all predicted protein sequences was performed using BLAST+ 2.3.0 searches with an E-value threshold of ≤1 × 10^−5^ and a minimum alignment coverage of 80% against six databases [66], including the Kyoto Encyclopedia of Genes and Genomes (KEGG), Clusters of Orthologous Groups (COG), NCBI non-redundant protein database (NR), Swiss-Prot, Gene Ontology (GO), and Pfam.

### 3.3. Phylogenetic and Clustering Analyses

We constructed a phylogenetic tree to assess the relatedness of our one food-related ST398-MRSA-Vc isolate and 134 previously published genome sequences from different geographic areas and sources (Supplementary Table S2) using single-copy core orthologs. These 134 sequences include genomes of 47 human-associated, 8 environmental, 29 food, and 50 animal (swine, *n* = 27; bovine, *n* = 12; other animals, *n* = 11) *S. aureus* isolates, comprising 27 MRSA of SCC*mec*Vc, 66 MRSA of other SCC*mec* types, and 41 MSSA isolates. Trees were generated under a maximum likelihood optimality criterion using FastTree (http://www.microbesonline.org/fasttree/, accessed on 28 September 2025), and annotated using iTOL (https://itol.embl.de). A further single-nucleotide polymorphism (SNP)-based analysis at the whole-genome level was performed on 12 closely related strains within the subclade containing strain NPREF115. The phylogenetic relatedness was evaluated with the CSI phylogeny 1.4 (available at https://cge.food.dtu.dk/services/CSIPhylogeny/, accessed on 16 October 2025) using default parameters [67], except for the minimum distance between SNP option, which was disabled. The SNP matrix and phylogenetic trees of the most relevant clones were constructed using the NPREF115 genome as a reference. The heatmap was plotted by https://www.bioinformatics.com.cn (last accessed on 18 October 2025), an online platform for data analysis and visualization [68].

### 3.4. SCCmec, MLST Typing, and Determination of Profiles of Virulence and Resistance Genes

By employing the SCC*mec*Finder 1.2 tool from the Center for Genomic Epidemiology (http://www.genomicepidemiology.org/, last accessed on 6 August 2025), with the criteria set as Identity ≥ 90% and minimum length ≥ 60%, the SCC*mec* types of the strains were determined. Additionally, sequence types were obtained using the website (https://pubmlst.org/). CARD database was used for antimicrobial gene identification (Identity ≥ 80%; Coverage ≥ 70%; E-value ≤ 1 × 10^−5^) [25], and VFDB (Identity ≥ 90%; Coverage ≥ 90%; E-value ≤ 1 × 10^−5^) for virulence factor determination [69].

### 3.5. Whole-Genome Alignment Analysis of Closely Related Strains

Comparative genomic analysis was performed using BRIG v0.95, with the complete genome of strain NPREF115 serving as the reference. Closely related strains identified from the phylogeny based on single-copy orthologs were included to visualize regions of genomic conservation and divergence. Regions exhibiting presence/absence variation were further examined for MGEs.

In this study, MGEs were operationally defined as genomic regions corresponding to SCC*mec*, prophages, genomic islands, ISs, and transposons. Genomic islands were predicted using IslandPath-DIMOB v1.0.0 and Islander v1.2 with default settings. Prophage regions were identified using PHASTER (https://phaster.ca, accessed on 7 December 2025) [70], and only regions classified as “intact” were considered for further analysis. Insertion sequences were detected using ISEScan v1.7.2.3 with default parameters and cross-validated against the ISfinder database (https://www-is.biotoul.fr) [71]. Putative transposable elements were identified using TransposonPSI (E-value cutoff ≤ 1 × 10^−5^).

### 3.6. Analysis of the Genetic Context of Mobile Genetic Elements

The prophage genomic map was generated using Proksee (https://proksee.ca/, accessed on 3 November 2025) [72]. A comparison of the genetic context was generated using Easyfig 2.2.5.

## 4. Conclusions

In this study, we provide the first genomic characterization of an ST398-MRSA-Vc isolate from RTE food in China. Strain NPREF115 exhibits a livestock-associated genetic profile, featuring a conserved core genome together with a dynamic mobilome enriched in MGEs. Notably, the presence of a chromosomally integrated transposon (Tn*560*) stabilizing a multidrug resistance gene cluster, along with metabolic determinants such as the 5-oxoprolinase gene that may facilitate adaptation to hyperosmotic food-associated environments, underscores the adaptive potential of this strain in the food production context. Although the strain lacks human-specific immune evasion determinants (e.g., the IEC cluster), its occurrence in RTE products highlights the pork supply chain as a potential interface for LA-MRSA dissemination. From a One Health perspective, the principal concern lies not in direct infection risk but in the potential for MGE-mediated horizontal transfer of AMR to humans. These findings underscore the need for large-scale and longitudinal surveillance of both domestic RTE foods and imported livestock products to better determine the prevalence and transmission dynamics of this high-risk lineage in China.

## Data Availability

All data are included in this study either in the manuscript or in the Supplementary Materials. Genome sequences can be found under NCBI GenBank (ID: CP187946 and CP187947). Source data are provided with this paper.

## Supplementary Materials

The following supporting information can be downloaded at: https://www.mdpi.com/article/10.3390/antibiotics15030314/s1, Figure S1: Circular map of MRSA isolate NPREF115; Figure S2: Upset plot of genes identified in NPREF115 and its phylogenetically related isolates; Figure S3: Circular genome map of prophage NPREF115-1. Table S1: Characteristics of MRSA isolate NPREF115; Table S2: Metadata for 135 isolates publicly available on NCBI; Table S3: ANI values between NPREF115 and phylogenetically related ST398 isolates; Table S4: Summary of mobile genetic elements identified in NPREF115.

## Abbreviations

The following abbreviations are used in this manuscript:

AMR	Antimicrobial resistance
ABC-F	ATP-binding cassette F
CFSA	China National Center for Food Safety Risk Assessment
COG	Clusters of Orthologous Groups
GO	Gene Ontology
HGT	Horizontal gene transfer
ICE	Integrative and conjugative element
IEC	Immune evasion cluster
IS	Insertion sequences
KEGG	Kyoto Encyclopedia of Genes and Genomes
LA-MRSA	Livestock-associated methicillin-resistant *Staphylococcus aureus*
MGE	Mobile genetic element
MRSA	Methicillin-resistant *S. aureus*
MSSA	Methicillin-susceptible *S. aureus*
NR	NCBI non-redundant protein database
ORFs	Open reading frames
RTE	Ready-to-eat
SaPIs	*Staphylococcal* pathogenicity islands
SCC*mec*	*Staphylococcal* cassette chromosome *mec*
SNP	Single-nucleotide polymorphism
ST	Sequence type
TAA	Trimeric autotransporter adhesin
Tn	Transposons
T4SS	Type IV Secretion System
