# Supplementary material for: Evolutionary and Mobile Genetic Element Analysis of a Multidrug-Resistant ST398-MRSA-Vc Isolate from Ready-to-Eat Pork Products
Source: Antibiotics (Basel). 2026 Mar 19;15(3):314. doi: 10.3390/antibiotics15030314 (PMC13024109; doi:10.3390/antibiotics15030314)
Supplement: Supplementary file 1 [file antibiotics-15-00314-s001.zip › supplement_materials.pdf]

Supplementary Material:

Evolutionary and Mobile Genetic Element Analysis of a Multidrug-Resistant ST398-MRSA-Vc Isolate from Ready-to-Eat Pork Product

Jinqi Wan [1a], Xiaoru Wang [1b], Kaifen Wang [b], Qiuyi Feng [b], Ruihua Yuan [b], Xiaojing Qi [b], Yidong Lai [b,*] and He Yan [a,c,*]

[a] College of Food Science and Engineering, South China University of Technology, Guangzhou, Guangdong 510640, China

[b] Dongguan Quality Supervision and Testing Center, Dongguan 523808, China

[c] Guangdong Province Key Laboratory for Green Processing of Natural Products and Product Safety, Guangzhou 510640, China

[1]Jinqi Wan and [1]Xiaoru Wang contributed equally.

*Corresponding author

Tel. and Fax: +86-20-87113848(He Yan); +86-0769-23071111(Yidong Lai)

Email: yanhe@scut.edu.cn (He Yan); lyd@gddqt.com(Yidong Lai)

Email addresses the following:

Jinqi Wan: wchinchi87@163.com

Xiaoru Wang: wxru@gddqt.com

Kaifen Wang: wkf@gddqt.com

Qiuyi Feng: fqy@gddqt.com

Ruihua Yuan: yrh@gddqt.com

Xiaojing Qi: qxj@gddqt.com

Yidong Lai: lyd@gddqt.com

He Yan: yanhe@scut.edu.cn

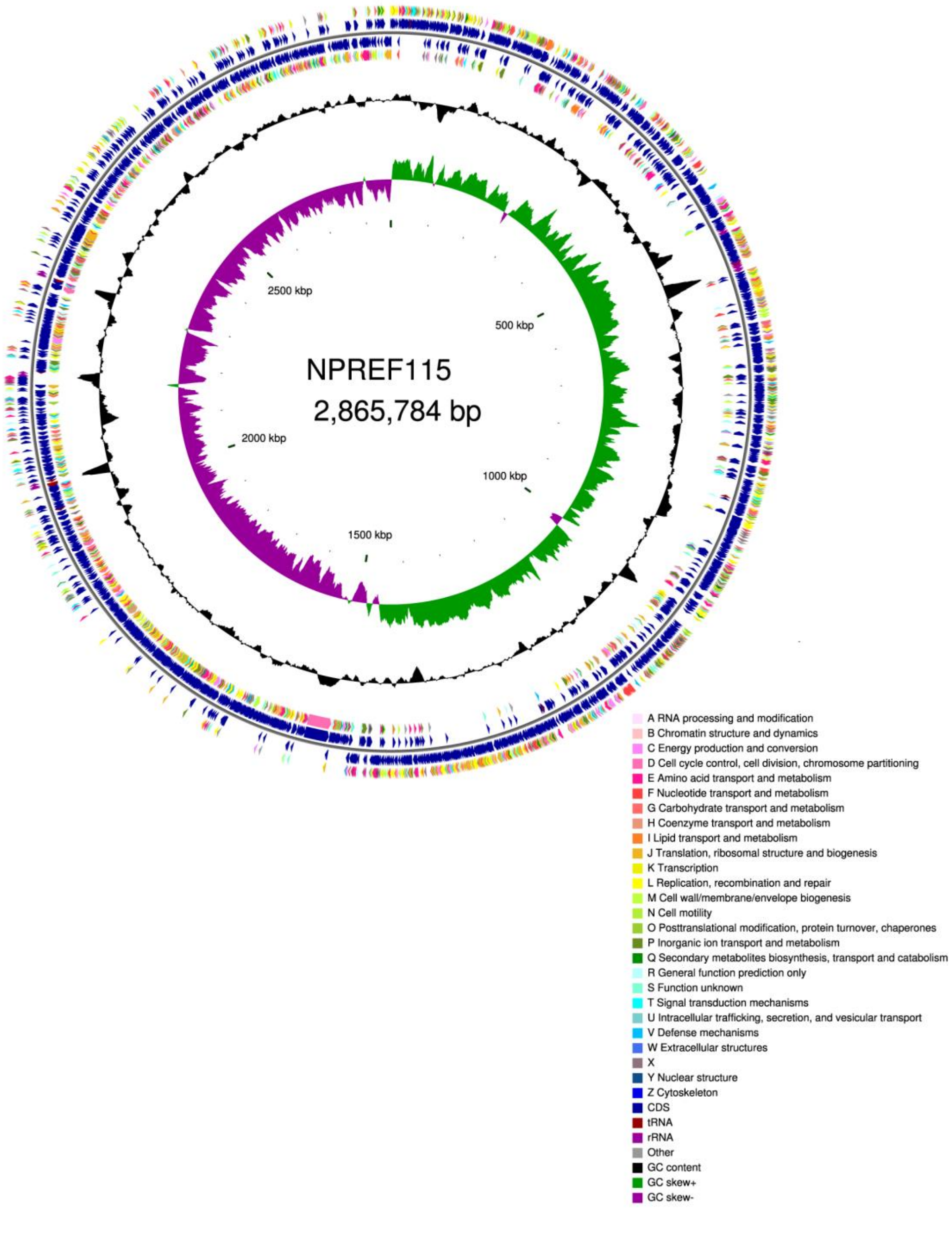

Figure.S1. Circular map of MRSA isolate NPREF115. From inside to outside, the first circle represents the genome size of NPREF115; the second circle represents the GC skew; the third circle represents the GC content; the fourth circle and the seventh circle represent the COG of each CDS; the fifth and sixth circles represent the

position of CDS, tRNA, and rRNA on the genome. CDS, coding genes; COG, Clusters of Orthologous Groups; GC, DNA G+C; rRNA, ribosomal RNA; tRNA, transfer RNA.

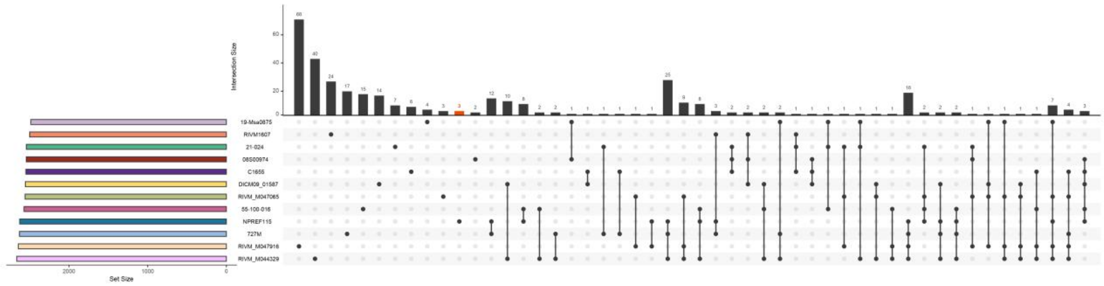

Figure.S2. Upset plot of genes identified in NPREF115 and its phylogenetically related isolates. Horizontal bar on left represents number of genes identified in each isolate. Dots and lines represent subsets of genes. Vertical histogram represents number of genes in each subset.

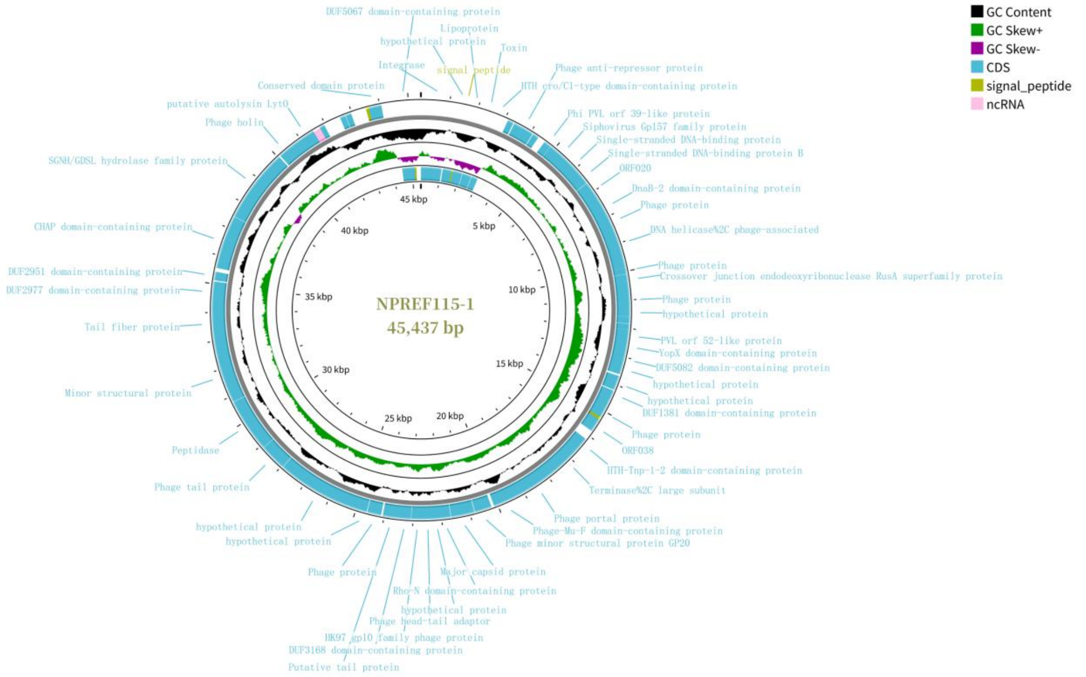

Figure.S3. Circular genome map of prophage NPREF115-1. From inside to outside, the first circle represents the genome size of prophage NPREF115-1; the second and

the fifth circles represent the position of CDS, ncRNA, and signal_peptide on the genome; the third circle represents the GC skew; the fourth circle represent the GC content. CDS, coding genes; GC, DNA G+C; ncRNA, non-coding RNA.

Table S1 Characteristics of MRSA isolate NPREF115.

	NPREF115
Location	Dongguan
Source	a ready-to-eat pork product
**Genotype**	
SCC*mec* type	Vc
MLST type	ST398
spa type	t011
Antibiotic resistance profile	oxacillin-cefoxitin-penicillin-nitrofurantoin-tetracycline-minocycline-clindamycin
**MICs(mg/L)**	
oxacillin	3
cefoxitin	12
penicillin	8
nitrofurantoin	64
tetracycline	96
minocycline	24
clindamycin	4
Antibiotic resistance genes	*mec*A, *bla*Z, *ant(9)*-Ia, *lsa*(E), *lnu*(B), *tet*(K), *tet*(M)

Table S2 Metadata for 135 isolates publicly available on NCBI. 135 isolates comprises four data sets (our one food-related ST398-MRSA-Vc isolate and 134 previously published genome sequences of *S. aureus* isolates). These 134 sequences include genomes of 47 human-associated, 8 environmental, 29 food, 50 animals (swine, n=27; bovine, n=12; other animals, n=11) *S. aureus* isolates, comprising 27 MRSA of SCC*mec*Vc, 66 MRSA of other SCC*mec* types and 41 MSSA isolates.

Isolate	Geographic region	Host	MRSA or MSSA	SCC*mec* Type	Sequence types	Genbank Accession
**food-related ST398-MRSA-Vc isolate sequenced in the present study**						
NPREF115	China	food	MRSA	Vc(5C2&5)	ST398	CP187946
**human-associated, environmental, food, animals *S. aureus* isolates in 134 previously sequenced genomes**						
ST398-MRSA-Vc isolates in 134 previously sequenced genomes						
LA-MRSA ST398	Canada	chicken	MRSA	Vc(5C2&5)	ST398	CP013218.1
55-100-016	Denmark	swine	MRSA	Vc(5C2&5)	ST398	CP076839.1
21-024	Switzerland	bovine	MRSA	Vc(5C2&5)	ST398	CP128389.1
ISU926	USA	swine	MRSA	Vc(5C2&5)	ST398	CP017091.1
08S00974	Germany	swine	MRSA	Vc(5C2&5)	ST398	CP020019.1
GDC6P096P	China	swine	MRSA	Vc(5C2&5)	ST398	CP065194.1
DG29	Germany	swine	MRSA	Vc(5C2&5)	ST398	CP172432.1
55-100-003	Denmark	swine	MRSA	Vc(5C2&5)	ST398	CP076843.1
PTDrAP2	Australia	swine	MRSA	Vc(5C2&5)	ST398	CP029172.1
21-074	Switzerland	bovine	MRSA	Vc(5C2&5)	ST398	CP128388.1
C1655	Spain	swine	MRSA	Vc(5C2&5)	ST398	JIZQ00000000.1
11P8	Netherlands	swine	MRSA	Vc(5C2&5)	ST398	JJDL00000000
11P4	Netherlands	swine	MRSA	Vc(5C2&5)	ST398	JJDM00000000
DICM09_01587	Spain	capra pyrenaica	MRSA	Vc(5C2&5)	ST398	JIYR00000000
Tur-15	Austria	chicken	MRSA	Vc(5C2&5)	ST398	JIWB00000000
C2706	Spain	swine	MRSA	Vc(5C2&5)	ST398	JIZJ01
5_3949	Germany	human	MRSA	Vc(5C2&5)	ST398	LT992462.1
RIVM_M047916	Netherlands	human	MRSA	Vc(5C2&5)	ST398	CP096535.1
RIVM_M047065	Netherlands	human	MRSA	Vc(5C2&5)	ST398	CP096539.1
RIVM_M044329	Netherlands	human	MRSA	Vc(5C2&5)	ST398	CP096540.1
RIVM_M083782	Netherlands	human	MRSA	Vc(5C2&5)	ST398	CP096532.1
RIVM1607	Netherlands	human	MRSA	Vc(5C2&5)	ST398	CP013619.1
RIVM_M084986	Netherlands	human	MRSA	Vc(5C2&5)	ST398	CP096528.1
S0385	Netherlands	human	MRSA	Vc(5C2&5)	ST398	AM990992.1
727M	Bulgaria	human	MRSA	Vc(5C2&5)	ST398	CP129560.1
RIVM_M085090	Netherlands	environment	MRSA	Vc(5C2&5)	ST398	CP096522.1
Other human-associated, environmental, food, animals *S. aureus* isolates in 134 previously sequenced genomes						
SA_9	China	food	MSSA	-	ST2990	Awaiting processing in NCBI
SA_12	China	food	MSSA	-	ST2250	Awaiting processing in NCBI
SA_14	China	food	MSSA	-	ST2250	Awaiting processing in NCBI
SA_19	China	food	MSSA	-	ST2250	Awaiting processing in NCBI
SA_27	China	food	MSSA	-	ST2990	Awaiting processing in NCBI

SA_42	China	food	MSSA	-	ST8952	Awaiting processing in NCBI
SA_44	China	food	MSSA	-	ST630	Awaiting processing in NCBI
SA_69	China	food	MSSA	-	ST2250	Awaiting processing in NCBI
SA_82	China	food	MSSA	-	ST15	Awaiting processing in NCBI
SA_84	China	food	MSSA	-	ST2250	Awaiting processing in NCBI
SA_86	China	food	MSSA	-	ST2250	Awaiting processing in NCBI
SA_96	China	food	MSSA	-	ST1	Awaiting processing in NCBI
SA_98	China	food	MSSA	-	ST1	Awaiting processing in NCBI
SA_H97	China	food	MSSA	-	ST7	Awaiting processing in NCBI
M22.2	Thailand	bovine	MRSA	V(5C2)	ST398	CP172401.1
PCFA-221	South Korea	swine	MRSA	V(5C2&5)	ST541	CP035003.1
LA31	Czech Republic	equus caballus	MRSA	IVa(2B)	ST398	CP144271.1
SA1428	USA	bovine	MSSA	-	ST6715	CP048431.1
GD4SA108-1	China	swine	MRSA	XII(9C2)	ST398	CP090375.1
CUVET17-1695	Thailand	canine	MRSA	Vb(5C2&5)	ST398	CP119710.1
PJFE-503	South Korea	swine	MRSA	XII(9C2)	ST398	CP049976.1
ST5477	Tanzania	bovine	MSSA	-	ST5477	CP135966.1
23EV612	New Zealand	bovine	MSSA	-	ST1	CP160024.1
M51	China	swine	MRSA	IVc(2B)	ST1516	CP030137.1
O82	France	sheep	MSSA	-	ST2011	CP038819.1
V315	Germany	duck	MSSA	-	ST9030	CP138362.1
NX-T55	China	swine	MRSA	XII(9C2)	ST9	CP031839.1
Z35	China	bovine	MRSA	XII(9C2)	ST9	CP062155.1
strain1	India	shrimp	MRSA	V(5C2&5)	ST772	CP102576.2
17-21	Russia	bovine	MSSA	-	ST97	CP126627.1
13	Brazil	bovine	MSSA	-	ST1	CP031265.1
IVB6170	Kenya	camel	MSSA	-	ST3573	CP094783.1
M48	China	swine	MRSA	III(3A)	ST239	CP030138.1
M19060305	South Korea	bovine	MRSA	IVa(2B)	ST22	CP142600.1
BSAR111_2	Denmark	sheep	MRSA	XI(8E)	ST130	CSBB00000000
BSAR202	United Kingdom	canine	MRSA	IV(2B)	ST22	CGHI00000000
M3	China	swine	MRSA	XII(9C2)	ST9	MWRY00000000
GKP136-11	United	bovine	MRSA	XI(8E)	ST130	FMPJ00000000

	Kingdom					
Rd.3	Germany	bovine	MRSA	IVa(2B)	ST398	JIWZ01
GD2010-102	Netherlands	swine	MRSA	IVa(2B)	ST398	JIYK01
43	China	swine	MSSA	-	ST1	Awaiting processing in NCBI
130	China	swine	MRSA	III(3A)	ST239	Awaiting processing in NCBI
57	China	swine	MSSA	-	ST7	Awaiting processing in NCBI
92	China	swine	MSSA	-	ST188	Awaiting processing in NCBI
107	China	swine	MSSA	-	ST7	Awaiting processing in NCBI
125	China	swine	MSSA	-	ST7	Awaiting processing in NCBI
M6	China	swine	MRSA	XII(9C2)	ST9	NCQK00000000
S57	China	swine	MRSA	XII(9C2)	ST9	CP030136.1
PCFH-226	South Korea	human	MRSA	V(5C2&5)	ST541	CP035005.1
RIVM3897	Netherlands	human	MRSA	V(5C2)	ST398	CP013621.1
CC239-MRSA-III	Trinidad and Tobago	human	MRSA	III(3A)	ST239	CP127019.1
MRSA-ATCC43300	Belgium	human	MRSA	II(2A)	ST39	CP123097.1
SA_0_0_1	Australia	human	MRSA	II(2A)	ST39	CP178839.1
PNID0137	South Korea	human	MSSA	-	ST977	CP071594.1
C257	Australia	human	MSSA	-	ST39	CP127794.1
19-Msa0875	Switzerland	human	MSSA	-	ST398	CP047646.1
CBTW2018367	China	human	MRSA	V(5C2)	ST1232	CP184559.1
2010C08-173	China	human	MRSA	Vb(5C2&5)	ST398	CP131661.1
CBTW2018043	China	human	MRSA	V(5C2&5)	ST1232	CP184565.1
BDH17	South Korea	human	MRSA	V(5C2&5)	ST1232	AP024315.1
19-00012	Germany	human	MRSA	IVa(2B)	ST398	CP125863.1
RIVM_M084526	Netherlands	human	MRSA	IVa(2B)	ST398	CP096530.1
THI2018-120	Japan	human	MRSA	V(5C2&5)	ST1232	AP024311.1
08BA02176	Canada	human	MRSA	V(5C2)	ST398	CP003808.1
MRSA-AMRF5	India	human	MRSA	V(5C2)	ST772	CP062467.1
N1195	Japan	human	MRSA	V(5C2&5)	ST1232	AP024313.1
2012-3	China	human	MRSA	V(5C2)	ST398	CP021178.1
SW-MRSA57	Switzerland	human	MRSA	I(1B)	ST228	CP122485.1
18082	China	human	MRSA	V(5C2)	ST398	CP041633.1
17Gst354	Switzerland	human	MRSA	IVa(2B)	ST398	CP073065.1
GE-MRSA18	Germany	human	MRSA	IVc(2B)	ST8	CP123151.1
RIVM_M087195	Netherlands	human	MRSA	IVa(2B)	ST398	CP096526.1
263	China	human	MSSA	-	ST22	CP138881.1

Sau38	Kenya	human	MRSA	Vc(5C2&5)	ST753	CP141477.1
MRSA_16_48b	Norway	human	MRSA	IVa(2B)	ST22	CP172293.1
MRSA-WC000	USA	human	MRSA	IV(2B)	ST5	CP092567.1
C70	Australia	human	MRSA	IVa(2B)	ST22	CP127716.1
MRSA252	United Kingdom	human	MRSA	II(2A)	ST36	BX571856.1
N08CSA36	China	human	MRSA	XII(9C2)	ST9	CP119571.1
JKD6159	USA	human	MRSA	IVa(2B)	ST93	CP002114.3
T0131	China	human	MRSA	III(3A)	ST239	CP002643.1
ER06690.3	USA	human	MRSA	VI(4B)	ST1	CP052008.1
FORC_027	South Korea	human	MRSA	II(2A)	ST5	CP012692.1
CA15	Colombia	human	MRSA	IVa(2B)	ST8	CP007674.1
MRSA_16_Hy	Norway	human	MRSA	IVa(2B)	ST22	CP172277.1
TSAR01	China	human	MRSA	V(5C2&5)	ST9	NADA00000000
LA52	Czech Republic	environment	MRSA	IVa(2B)	ST398	CP144270.1
CFSAN007894	USA	environment	MSSA	-	ST5	CP045866.1
24EBSta0529	Korea	environment	MSSA	-	ST59	CP166872.1
WHC07	China	environment	MSSA	-	ST9	CP077757.1
IPLA15	Spain	environment	MSSA	-	ST1	CP134618.1
JB2E56	South Korea	environment	MRSA	XII(9C2)	ST398	CP170579.1
Azir	Taiwan	environment	MRSA	pseudo-SCC *mec* element	ST9	CP113016.1
10-5-1	South Korea	food	MSSA	-	ST5870	CP134882.1
B6-55A	USA	food	MSSA	-	ST398	CP042110.1
B3-14B	USA	food	MSSA	-	ST398	CP042003.1
13-ST00660	Germany	food	MRSA	IVa(2B)	ST398	CP011874.1
14_ST00667	Germany	food	MRSA	IVa(2B)	ST398	CP011873.1
63	South Korea	food	MSSA	-	ST15	CP134068.1
FORC_001	South Korea	food	MSSA	-	ST30	CP009554.1
FORC_039	South Korea	food	MRSA	IVa(2B)	ST188	CP015817.1
FORC_040	South Korea	food	MRSA	IVc(2B)	ST72	CP016398.1
NV_1	China	food	MRSA	XV(7A)	ST5	CP080249.1
NT_611	China	food	MRSA	pseudo-SCC *mec* element	ST88	CP080251.1
MFDS1022333	South Korea	food	MSSA	negative	ST6	CP138576.1
2868B2	China	food	MRSA	XII(9C2)	ST9	CP060141.1
NT_8	China	food	MRSA	pseudo-SCC *mec* element	ST3686	CP080222.1
12S01032	Germany	food	MRSA	IVa(2B)	ST398	CP011877.1

Table S3 ANI values between NPREF115 and phylogenetically related ST398 isolates.

Isolate ID	Host origin	Country	ANI (%) vs NPREF115ANI (%) vs NPREF115
08S00974	Swine	Germany	99.9175
19-Msa0875	Human	Switzerland	99.8976
21-024	Bovine	Switzerland	99.9083
55-100-016	Swine	Denmark	99.9687
727M	Human	Bulgaria	99.8958
C1655	Swine	Spain	99.9105
DICM09_01587	Capra pyrenaica	Spain	99.8862
RIVM1607	Human	Netherlands	99.9035
RIVM_M044329	Human	Netherlands	99.8858
RIVM_M047065	Human	Netherlands	99.892
RIVM_M047916	Human	Netherlands	99.8001

Table S4 Summary of mobile genetic elements identified in NPREF115.

MGE ID	Type	Genomic Location (bp)	Size (bp)	ARGs	Virulence Genes
SCC*mec* Vc	SCCmec	33751-80304	46553	*mecA*, *tet*(K)	none
Prophage 1	Prophage	872123-917560	45437	none	none
Prophage 2	Prophage	1606024-1650802	44778	none	none
IS*21*	Insertion sequence	82765-84961	2196	none	none
IS*Sau1*	Insertion sequence	1363299-1364357	1058	none	none
IS*Sau1*	Insertion sequence	2351163-2352251	1088	none	none
IS*Sau1*	Insertion sequence	2489135-2490192	1057	none	none
IS*Sau8*	Insertion sequence	2204689-2206185	1496	none	none
Transposon-1	Transposon	626595-627191	596	none	none
Transposon-2	Transposon	784442-784750	308	none	none
Transposon-3	Transposon	2765294-2765866	572	none	none
Tn*6009*	Transposon	1004558-1006446	1888	none	none
GI-1	Genomic island	332550-339802	7252	none	none
GI-2	Genomic island	435446-445302	9856	none	none
GI-3	Genomic island	682287-687742	5455	none	none
GI-4	Genomic island	771839-785833	13994	none	none
GI-5	Genomic island	1005857-1021747	15890	*tet*(M)	none
GI-6	Genomic island	1386331-1392435	6104	none	none
GI-7	Genomic island	1803238-1821212	17974	*ant(9)-Ia*, *lsa*(E), *lnu*(B)	none
GI-8	Genomic island	1979498-1991246	11748	none	none
GI-9	Genomic island	2101847-2112831	10984	*blaZ*	none
GI-10	Genomic island	2261812-2306883	45071	none	none
