# Supplementary figures and images for: Evolutionary and Mobile Genetic Element Analysis of a Multidrug-Resistant ST398-MRSA-Vc Isolate from Ready-to-Eat Pork Products
Source: Antibiotics (Basel). 2026 Mar 19;15(3):314. doi: 10.3390/antibiotics15030314 (PMC13024109; doi:10.3390/antibiotics15030314)

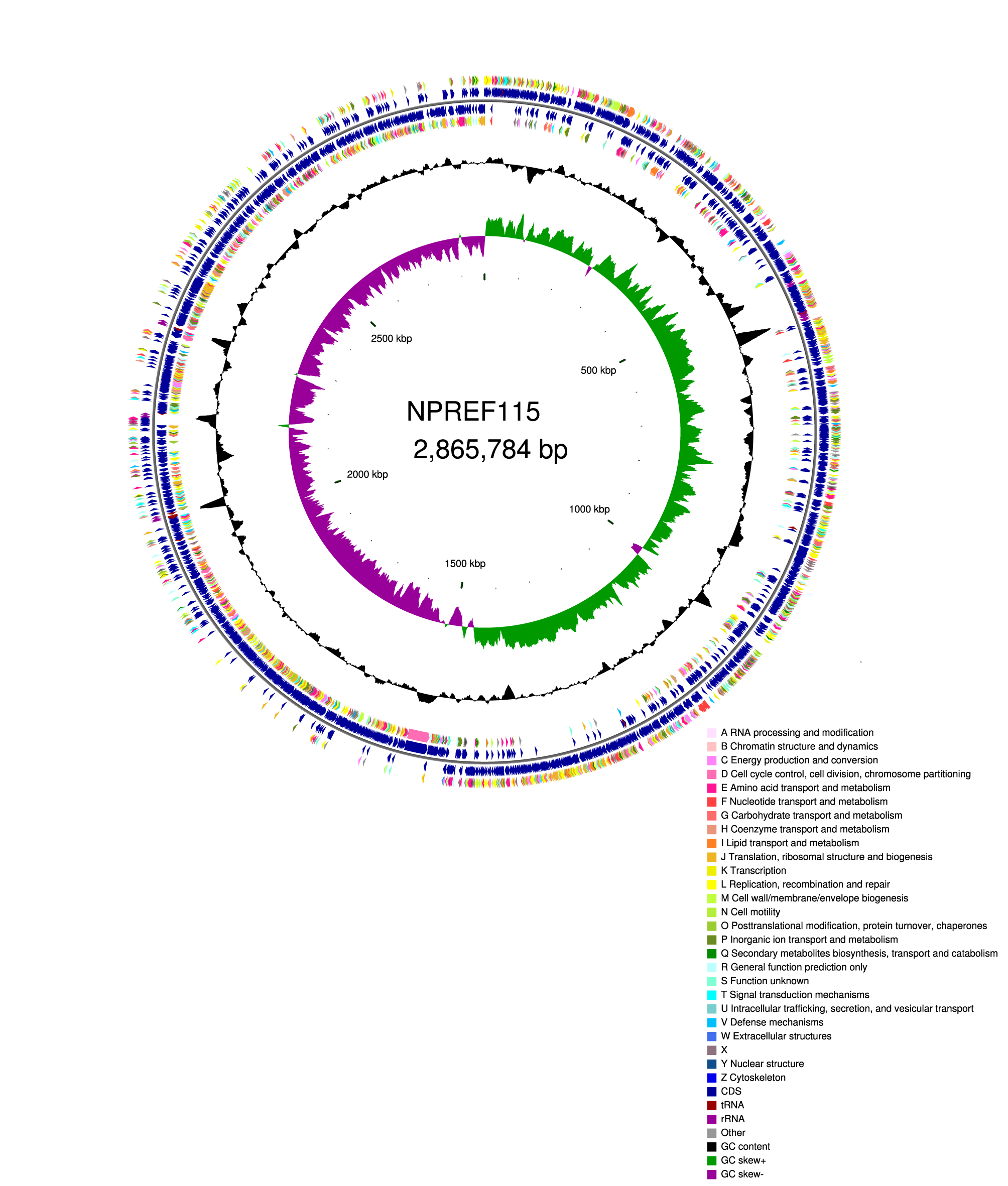

Supplement: Supplementary file 1 [file antibiotics-15-00314-s001.zip › Figure S1.tif]

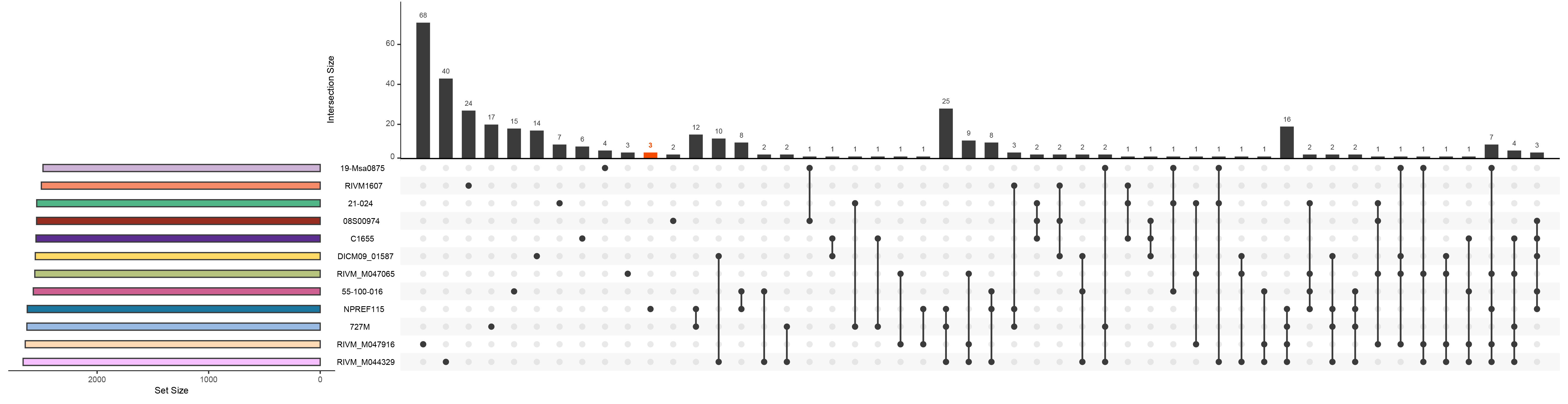

Supplement: Supplementary file 1 [file antibiotics-15-00314-s001.zip › Figure S2.png]

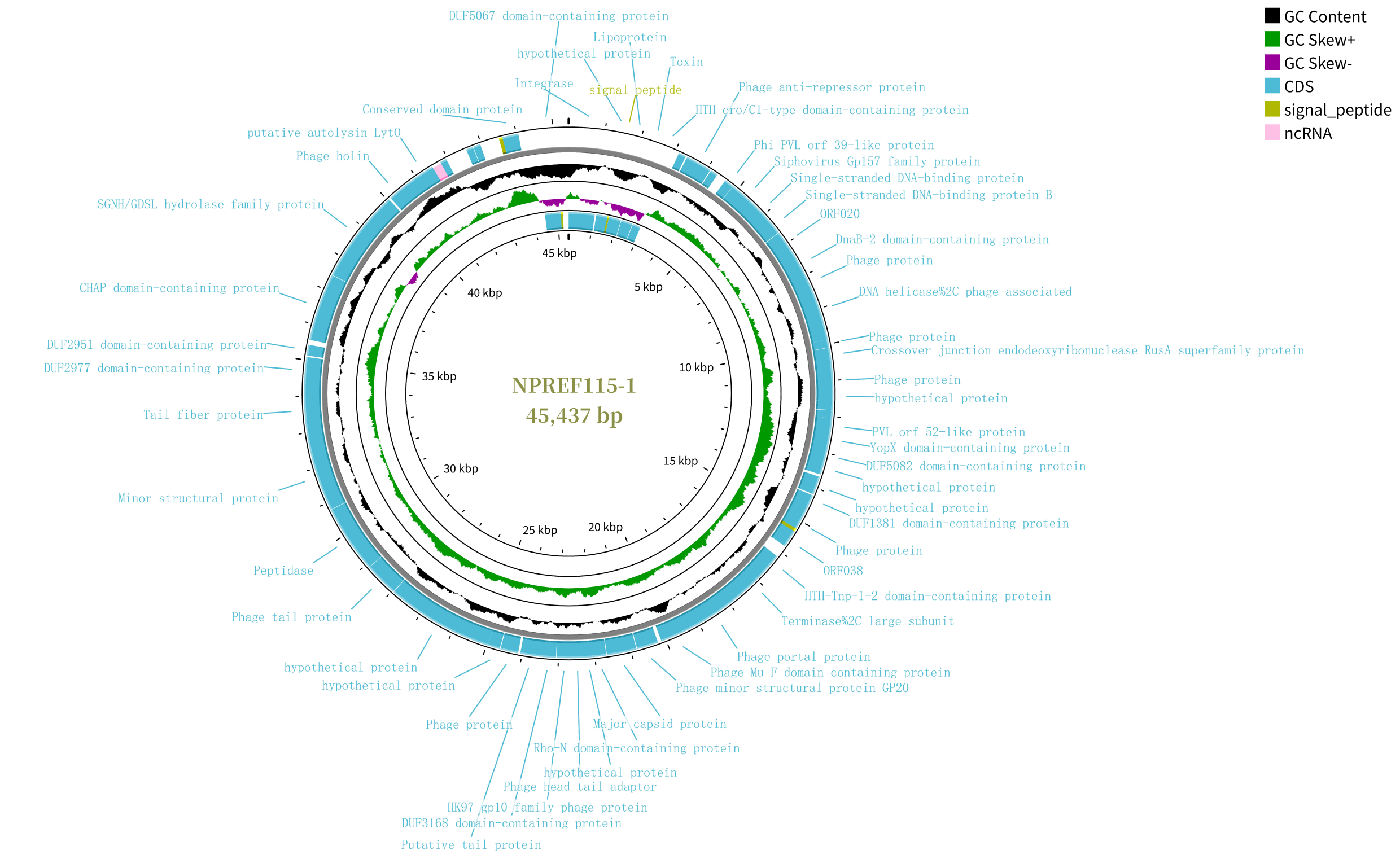

Supplement: Supplementary file 1 [file antibiotics-15-00314-s001.zip › Figure S3.tif]
